# Development and validation of a nomogram for predicting survival time and making treatment decisions for clinical stage IA NSCLC based on the SEER database

**DOI:** 10.3389/fmed.2022.972879

**Published:** 2022-12-21

**Authors:** Bingchen Xu, Ziming Ye, Lianxin Zhu, Chunwei Xu, Mingjian Lu, Qian Wang, Wang Yao, Zhihua Zhu

**Affiliations:** ^1^State Key Laboratory of Oncology in South China, Department of Thoracic Surgery, Collaborative Innovation Center for Cancer Medicine, Sun Yat-sen University Cancer Center, Guangzhou, China; ^2^Medical College of Nanchang University, Nanchang, China; ^3^Queen Mary University of London, London, United Kingdom; ^4^Department of Medical Oncology, Affiliated Jinling Hospital, Medical School of Nanjing University, Nanjing, China; ^5^Department of Radiology, Affiliated Cancer Hospital and Institute of Guangzhou Medical University, Guangzhou, Guangdong, China; ^6^Department of Respiratory Medicine, Jiangsu Province Hospital of Chinese Medicine, Affiliated Hospital of Nanjing University of Chinese Medicine, Nanjing, China; ^7^Department of Interventional Oncology, The First Affiliated Hospital, Sun Yat-sen University, Guangzhou, China

**Keywords:** nomogram, SEER, prognosis, NSCLC, treatment

## Abstract

**Background:**

The aim of this study was to establish and validate a nomogram model for accurate prediction of patients’ survival with T1aN0M0 none small cell lung cancer (NSCLC).

**Methods:**

The patients, diagnosed with the stage IA NSCLC from 2004–2015, were identified from the Surveillance, Epidemiology and End Results (SEER) database. The variables with a *P*-value < 0.05 in a multivariate Cox regression were selected to establish the nomogram. The discriminative ability of the model was evaluated by the concordance index (C-index). The proximity of the nomogram prediction to the actual risk was depicted by a calibration plot. The clinical usefulness was estimated by the decision curve analysis (DCA). Survival curves were made with Kaplan–Meier method and compared by Log–Rank test.

**Results:**

Eight variables, including treatment, age, sex, race, marriage, tumor size, histology, and grade were selected to develop the nomogram model by univariate and multivariate cox regression. The C-index was 0.704 (95% CI, 0.694–0.714) in the training set and 0.713 (95% CI, 0.697–0.728) in the test set, which performed significantly better than 8th edition AJCC TNM stage system (0.550, 95% CI, 0.408–0.683, *P* < 0.001). The calibration curve showed that the prediction ability of 3-years and 5-years survival rate demonstrated a high degree of agreement between the nomogram model and the actual observation. The DCA curves also proved that the nomogram-assisted decisions could improve patient outcomes.

**Conclusion:**

We established and validated a prognostic nomogram to predict 3-years and 5-years overall survival in stage IA NSCLC.

## Introduction

Lung cancer remains the leading cause of cancer death, causing 1.8 million deaths each year and accounting for 18% of deaths worldwide (1). The major histologic subtype of lung cancer is non-small cell lung cancer (NSCLC) (2). Early stage NSCLC can be easily clinically diagnosed due to the recent development of high-resolution thin-slice computerized tomography (CT) (3). However, due to the variety of treatments available for early stage NSCLC, the selection of suitable treatment for patients with different health conditions has always been problematic.

After the first prospective report presented by the Lung Cancer Study Group (LCSG), which in a randomized trial determined that limited resection results in a higher death rate and a higher locoregional recurrence rate, lobectomy with mediastinal lymph node dissection became the standard treatment for T1N0M0 NSCLC (4). However, several studies reported that survival after sublobar resection or ablation was similar to that after lobectomy. The possible reason for the observed differences could be that the LCSG study was based solely on the tumor-node-metastasis (TNM) staging system and ignored the influence of clinicopathologic features on patient prognosis. A recent study conducted in Japan that took tumor characteristics into consideration found that segmentectomy resulted in morbidity and mortality equal to that observed after lobectomy in patients with tumor diameter ≤2 cm and a C/T ratio >0.5 (5). Similarly, in JACS1303, a study in which baseline factors were also considered, it was demonstrated that wedge resection might be equivalent to lobectomy or segmentectomy in selected patients more than 80 years of age (6). Therefore, a new prognostic model that includes prognostic factors such as age, sex, and tumor characteristics is needed for clinical decision-making.

Nomograms have been used extensively as visualization prediction models. Many studies have confirmed that prediction models can predict overall survival in patients with liver cancer, prostate cancer and small cell lung cancer (7–9). However, to our knowledge, there is currently no similar nomogram model for clinical decision-making in early stage NSCLC. Therefore, in this research, we built an innovative nomogram model that utilizes the TNM system as well as other prognostic factors to improve the personalized risk staging system and facilitate decision-making regarding individual treatment.

## Materials and methods

### Patients

**FIGURE 1:**
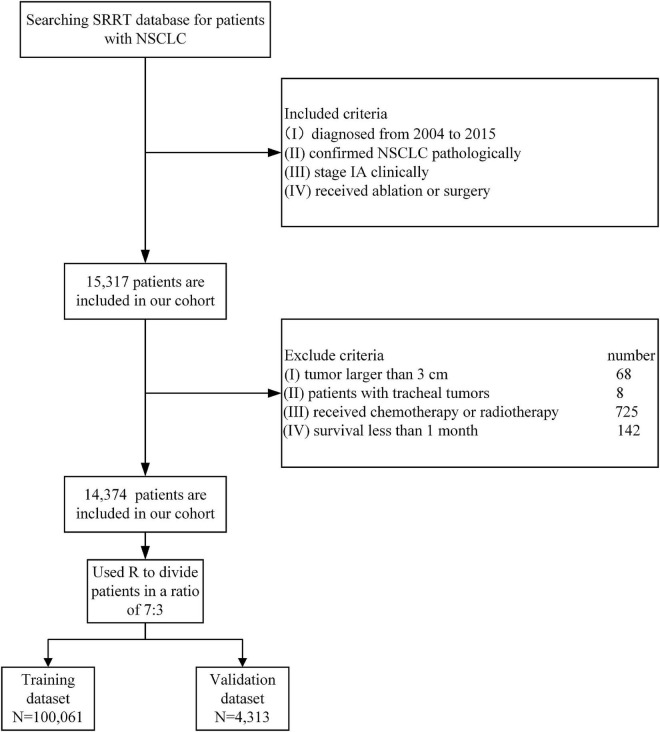
Flow-chart illustrating the steps to extract the case of NSCLC from SEER.

The Surveillance, Epidemiology and End Results (SEER) database was our source of population-based data for nomogram development and validation. The date of accession of the SEER database was 20 August 2021. The version of the database used in this study was SEER×STAT 8.3.9.2. A flow chart illustrating the methodology that was used to extract data on patients with stage IA NSCLC who were included in the SEER database during the period 2004–2015 is shown in Figure 1. The patient inclusion criteria were as follows: (I) Diagnosed with NSCLC between 2004 and 2015 according to ICD-O-3 codes 8,012, 8,046, 8,070–8,072, 8,140, 8,250, 8,255, 8,260, 8,480–8,481, 8,490, 8,550, 8,560, or 8,570; (II) clinically confirmed stage IA NSCLC based on the 8th edition of the TNM classification (10); (III) underwent ablation, wedge resection, segmentectomy or lobectomy; and (IV) only one primary tumor present. The exclusion criteria were as follows: (I) received radiotherapy, adjuvant chemotherapy, neoadjuvant chemotherapy or other systemic treatment; (II) had severe complications or died within 30 days after surgery; (III) lost to follow-up; and (IV) history of prior synchronous or metachronous malignancies. Data on 15,317 patients were extracted from the SEER database, and 14,374 of these patients were selected as our cohort. The sample split function of the “catools” package in R was used to divide the 14,374 samples into a training cohort (*N* = 10,061) and a validation cohort (*N* = 4,313) at a ratio of 7:3. The details and codes used in the selection are shown in Table 1.

**TABLE 1:** Details patient selection criteria with variable names used and their effect on sample size.

Step	Selection criteria	Code	Count
1	Select respiratory SEER data with schema–lung	{Site and Morphology.Site recode ICD-O-3/WHO 2008} = ‘ Lung and Bronchus’	972,941
2	Only patients with one primary	AND {Multiple Primary Fields.Sequence number} = ‘One primary only’	695,122
3	Year of diagnosis from 2004 to 2015	AND {Race, Sex, Year Dx, Registry, County.Year of diagnosis} = ‘2004’,‘2005’,‘2006’,‘2007’,‘2008’,‘2009’,‘2010’,‘2011’,‘2012’,‘2013’,‘2014’,‘2015’	436,736
4	SEER historic stage A = 1	AND {Stage–Summary/Historic.SEER historic stage A (1973–2015)} = ‘Localized’	65,092
5	Only patients with one malignant primary	AND {Multiple Primary Fields.First malignant primary indicator} = ‘Yes’	65,092
6	Select only patients with segmentectomy and wedge resection	AND {Therapy.RX Summ–Surg Prim Site (1998+)} = 12–13,15,21–22,33	26,687
7	Select only IA stage (AJCC 6th) from 2005 to 2009 or IA stage (AJCC 7th) from 2004 to 2015	AND {Stage–6th edition.Derived AJCC Stage Group, 6th ed (2004–2015)} = ‘IA’	16,970
8	Select Non-small cell lung cancer	AND {Site and Morphology.Histologic Type ICD-O-3} = 8,012,8,046,8,070–8,072,8,140,8,250,8,255,8,260,8,480–8,481,8,490,8,550,8,560,8,570	15,317
9	Select 1 mm ≤ tumor size ≤ 30 mm	AND {Extent of Disease.CS tumor size (2004–2015)} = 1–30,991–993	15,249
10	Exclude tracheal tumors	AND {Site and Morphology.Primary Site}! = 340	15,241
11	Exclude chemotherapy	AND {Therapy.Chemotherapy recode (yes, no/unk)}! = ‘Yes’	14,798
12	Exclude radiotherapy	AND {Therapy.RX Summ–Surg/Rad Seq} = ‘No radiation and/or cancer-directed surgery’	14,516
13	Exclude survivaltime less than 1 month	AND {Cause of Death (COD) and Follow-up.Survival months}! = 0	14,374

### Variables

In this study, we collected patient baseline, treatment model and follow-up information, including age, sex, race, marital status, laterality, primary site, tumor size, histologic type, grade, marital status, and survival time. Age was converted to a categorial variable in this study using a cut-off value of 75 years determined by R software. Tumor stage, which was also an ordered variable, was converted to a categorial variable. The treatment methods consisted of lobectomy, segmentectomy, wedge resection and ablation, and each patient received only one treatment. The primary endpoint of the study was OS calculated from the date of diagnosis to patient death or loss to follow-up.

### Nomogram model development

The model was established using the training set and verified using the validation set to reduce overfitting and upwardly biased estimates of performance. We used univariate Cox regression to select covariables associated with OS. Factors with *P*-values < 0.10 were entered into the multivariate Cox regression analysis. Multivariate Cox regression was then used to identify independent prognostic factors with *P*-values < 0.05. The prediction model was established based on the prognostic variables in the final model.

### Validation of the nomogram

A validation process was used to obtain unbiased estimates of the model’s performance and judge its applicability to different populations. First, we used bootstrapping, which was iteratively applied to randomly selected sample sets of the training cohort, to prevent overinterpretation. The discrimination ability of the prediction model was assessed by the concordance index (C-index). We then constructed calibration curves that we used to evaluate the accuracy of the model by comparing the predicted survival times and observed survival rates at 3 and 5 years. Last, we used decision curve analysis to assess whether nomogram-assisted decision-making could improve patient outcomes.

### Statistical analysis

Continuous variables were converted to categorical variables using the median as a cut-off value, and categorical variables are denoted as percentages. The χ^2^ test was used to analyze differences between two groups. Survival outcomes were evaluated by the Kaplan–Meier method and compared using the log-rank test. Univariate and multivariate Cox proportional hazards regression analyses were conducted to evaluate the strength of the association between OS and potential risk factors. All statistical analyses were conducted in R software (version 4.1.0).^1^ The R packages “gtsummary,” “dplyr,” “flextable,” “survival,” “catools,” “rms,” “crosstalk,” “dynnom,” “rsconnect,” “cvauc,” and “regplot” were used.

## Result

### Baseline characteristics of patients

This study included a total of 15,317 patients for whom data was available in the SEER database. The baseline characteristics of the patients are shown in Table 2. In the comparison between the training set and the testing set, none of the variables, including surgery (*P* = 0.656), age (*P* = 0.692), sex (*P* = 0.667), race (*P* = 0.990), marital status (*P* = 0.724), laterality (*P* = 0.733), site (*P* = 0.237), size (*P* = 0.812), histology (*P* = 0.381), and grade (*P* = 0.173), differed significantly. The median survival times were 114 months (range, 0–179 months) in the training dataset and 110 months in the testing dataset (range, 0–179 months). The 3-year OS rates for the training and testing datasets were 81.1% (95% CI, 80.4–81.9%) and 81.3% (95% CI, 80.1–82.4%), respectively. The 5-year OS rates for the training and testing datasets were 69.9% (95% CI, 69.0–70.8%) and 70.0% (95% CI, 68.6–71.4%), respectively.

**TABLE 2:** Characteristics of stage IA NSCLC patients from SEER database.

–	Training set	Test set	–
	*N* = 10,061^1^	*N* = 4,313^1^	*P*-value^2^
Surgery	–	–	0.656
Ablation	110 (1.1%)	40 (0.9%)	–
Wedge resection	2,156 (21%)	921 (21%)	–
Segmentectomy	583 (5.8%)	267 (6.2%)	–
lobectomy	7,212 (72%)	3,085 (72%)	–
Age	–	–	0.692
<75 year	7,389 (73%)	3,182 (74%)	–
≥75 year	2,672 (27%)	1,131 (26%)	–
Sex	–	–	0.667
Female	5,828 (58%)	2,481 (58%)	–
Male	4,233 (42%)	1,832 (42%)	–
Race	–	–	0.990
White	8,519 (85%)	3,648 (85%)	–
Black	805 (8.0%)	347 (8.0%)	–
Other	737 (7.3%)	318 (7.4%)	–
Marital status	–	–	0.724
Yes	5,602 (56%)	2,387 (55%)	–
No	4,459 (44%)	1,926 (45%)	–
Laterality	–	–	0.733
Left	4,103 (41%)	1,745 (40%)	–
Right	5,958 (59%)	2,568 (60%)	–
Primary site	–	–	0.237
Upper lobe	6,397 (64%)	2,729 (63%)	–
Middle lobe	434 (4.3%)	219 (5.1%)	–
Lower lobe	3,139 (31%)	1,329 (31%)	–
Other	91 (0.9%)	36 (0.8%)	–
T stage	–	–	0.812
T1a	1,172 (12%)	513 (12%)	–
T1b	5,314 (53%)	2,254 (52%)	–
T1c	3,575 (36%)	1,546 (36%)	–
Histologic type	–	–	0.381
SCC	2,398 (24%)	983 (23%)	–
ADC	7,060 (70%)	3,074 (71%)	–
Other	603 (6.0%)	256 (5.9%)	–
Grade	–	–	0.173
I/II	6,829 (68%)	2,870 (67%)	–
III/IV	2,604 (26%)	1,144 (27%)	–
Unknown	628 (6.2%)	299 (6.9%)	–

SCC, squamous cell carcinoma; ADC, adenocarcinoma.

^1^*n* (%); ^2^Pearson’s Chi-squared test; Fisher’s exact test.

### Selection of independent prognostic factors and establishment of a nomogram

**FIGURE 2:**
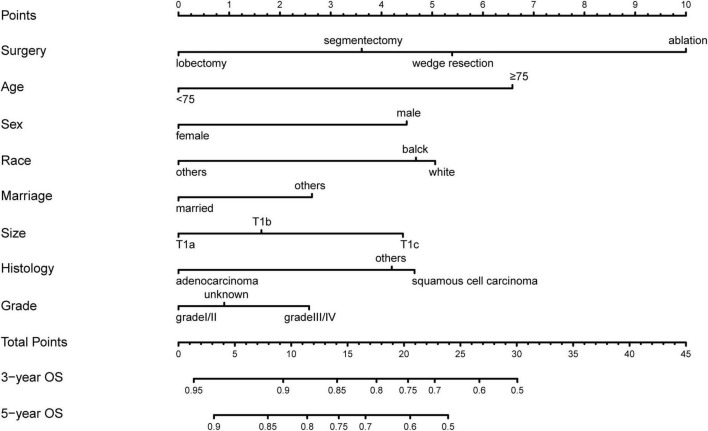
A total of 3 years- and 5 years- overall survival nomogram for patients with stage IA NSCLC.

The results of univariate analyses indicated that factors such as treatment (*P* < 0.001), age (*P* < 0.001), sex (*P* < 0.001), race (*P* < 0.001), marital status (*P* < 0.001), tumor size (*P* < 0.001), histology (*P* < 0.001), and grade (*P* < 0.001) were associated with patient prognosis (Table 3). The laterality and site of tumors were not independent risk factors (*P* = 0.071 and *P* = 0.130, respectively). In further analysis using multivariable Cox regression, treatment (*P* < 0.001), age (*P* < 0.001), sex (*P* < 0.001), race (*P* < 0.001), marital status (*P* < 0.001), tumor size (*P* < 0.001), histology (*P* < 0.001), and grade (*P* < 0.001) were identified as independent prognostic factors (Table 3). The above factors were used to develop a prediction model, which is virtually presented in Figure 2 in the form of a nomogram. It can be observed that treatment had the greatest impact on patient survival and that grade and marital status contributed moderately to survival. Each straight line in the nomogram represents a factor for which a corresponding number of points was assigned to a particular magnitude of the variable. Point scores were then cumulated for all the variables and located on the point scale in a way that reflected their influence on the outcome. Using the nomogram, it was easy to obtain a final risk score and predict the OS at 3 and 5 years for specific patients.

**TABLE 3:** Univariate and multivariate Cox regression of OS in stage IA NSCLC patients.

–	Univariate		–	Multivariate		–
	HR^1^	95% CI^2^	*P*-value	HR	95% CI	*P*-value
Treatment	–	–	–	–	–	–
Ablation	–	–		–	–	
Wedge resection	0.52	0.41, 0.64	<0.001	0.67	0.54, 0.84	<0.001
Segmentectomy	0.41	0.32, 0.52	<0.001	0.57	0.44, 0.73	<0.001
Lobectomy	0.30	0.24, 0.37	<0.001	0.41	0.33, 0.51	<0.001
Age	–	–	–	–	–	–
<75 year	–	–	–	–	–	–
≥75 year	2.06	1.94, 2.20	<0.001	1.82	1.70, 1.94	<0.001
Sex	–	–	–	–	–	–
Female	–	–		–	–	
Male	1.49	1.40, 1.58	<0.001	1.50	1.41, 1.60	<0.001
Race	–	–	–	–	–	–
White	–	–		–	–	
Black	0.97	0.87, 1.09	0.600	0.97	0.86, 1.08	0.600
Other	0.56	0.48, 0.65	<0.001	0.63	0.55, 0.73	<0.001
Marital status	–	–	–	–	–	–
Yes	–	–		–	–	
No	1.29	1.22, 1.38	<0.001	1.27	1.19, 1.35	<0.001
Laterality	–	–	–	–	–	–
Left	–	–	–	–	–	–
Right	0.95	0.89, 1.00	0.071	–	–	–
Primary site	–	–	–	–	–	–
Upper lobe	–	–	–	–	–	–
Middle lobe	0.89	0.76, 1.04	0.130	–	–	–
Lower lobe	0.97	0.91, 1.04	0.400	–	–	–
Other	1.01	0.74, 1.38	>0.9	–	–	–
T stage	–	–	–	–	–	–
T1a	–	–	–	–	–	–
T1b	1.14	1.03, 1.27	0.014	1.16	1.04, 1.29	0.006
T1c	1.51	1.35, 1.68	<0.001	1.49	1.34, 1.67	<0.001
Histologic type	–	–	–	–	–	–
SCC	–	–	–	–	–	–
ADC	0.53	0.49, 0.56	<0.001	0.66	0.61, 0.70	<0.001
Other	1.00	0.89, 1.12	>0.9	0.96	0.85, 1.08	0.500
Grade	–	–	–	–	–	–
I/II	–	–	–	–	–	–
III/IV	1.55	1.45, 1.65	<0.001	1.26	1.18, 1.35	<0.001
Unknown	1.24	1.10, 1.41	<0.001	1.09	0.95, 1.24	0.200

^1^HR, hazard ratio; ^2^CI, confidence interval.

### Calibration of the nomogram prediction model

**FIGURE 3:**
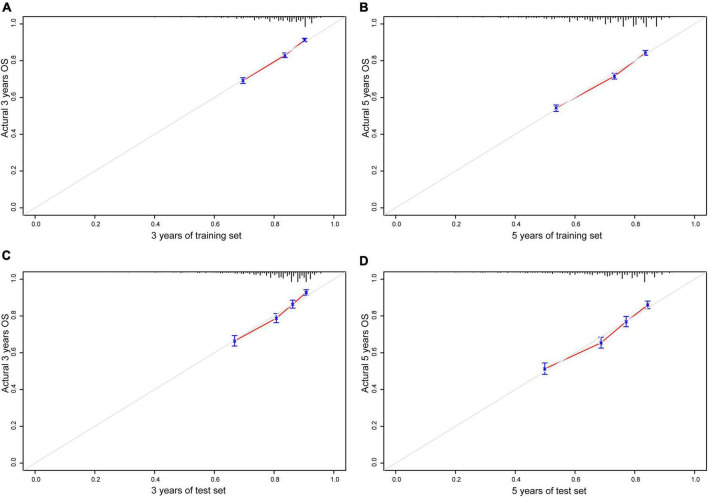
Calibration plots of nomogram model. **(A)** A total of 3 years of training set; **(B)** 5 years of training set; **(C)** 3 years of test set; **(D)** 5 years of test set; gray line, actual observation; red line, prediction survival rate.

**FIGURE 4:**
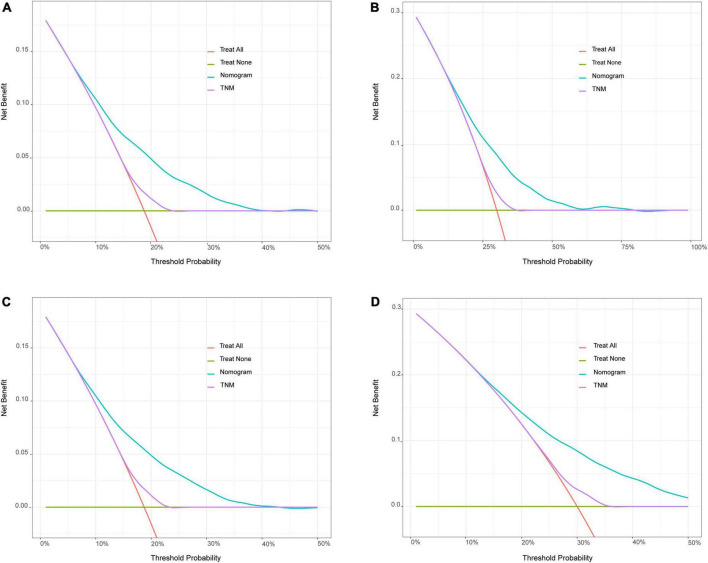
Decision curve analysis (DCA) for the nomogram model and the 8th edition AJCC TNM staging system. **(A)** Comparison of 3 years of DCA in training set. **(B)** Comparison of 5 years of DCA in training set. **(C)** Comparison of 3 years of DCA in test set. **(D)** Comparison of 5 years of DCA in test set. The X-axis referred to the threshold probabilities, defined as the minimum probability of disease at which further intervention would be warranted. The Y-axis referred to the net benefit. When the death rate exceeded a certain value, the clinician should start treatment (intervention) for patients. After starting the treatment, the patients who actually will die would be benefited from the treatment (positive predictivity), and those who will not die would be harmed by the treatment (false positive rate). The net benefit = positive predictivity–false positive rate. The green horizontal line represents none of the patients received intervention and the other oblique orange lines represent all patients have been treated.

Bootstrapping was used with 1,000 repetitions, resampling 3,000 samples a time to prevent data overinterpretation and obtain a relatively unbiased estimate of the performance of the model. The C-indices of the nomogram were 0.704 (95% CI, 0.694–0.714) and 0.713 (95% CI, 0.697–0.728) in the training cohort and the test cohort, respectively, reflecting the adequacy of the model in distinguishing between patients who received different treatments. In contrast, the C-indices for OS estimates based on staging according to the eighth edition of the AJCC TNM staging system were 0.550 (95% CI, 0.408–0.683) in the training set and 0.548 (95% CI, 0.401–0.672) in the test set. The calibration curves for the model of 3- and 5-year OS are shown in Figure 3 and demonstrate the closeness of the nomogram’s predictions to the actual observations. Additionally, the results remained consistent in the test set. It is shown in Figure 4 that DCA exhibited great positive net benefits among all the threshold probabilities at different time points, indicating the favorable potential clinical effect of the predictive model.

## Discussion

Stage I NSCLC represents a very early stage of lung cancer. Due to the high heterogeneity of NSCLC and the development of CT technology for diagnosis, the treatment choices that are made for different patients show considerable discrepancy (11). With the continuous progress in precision medicine and individualized treatment, the TNM staging system, which is based solely on anatomical classification, is no longer sufficiently accurate to be clinically useful. Therefore, it is urgently necessary to develop a new prediction model that can be used to assist clinicians in the selection of suitable treatment strategies based on routinely measured clinicopathological variables. In this study, a predictive nomogram based on the patients’ pretreatment clinicopathological characteristics was established, and its accuracy in predicting the prognosis of patients with stage IA NSCLC was verified.

Many clinical factors affect the survival of patients with NSCLC (12, 13). Notably, treatment management is an important prognostic factor in lung cancer patients. Whitson et al. reported that lobectomy conferred superior overall (*P* < 0.0001) and cancer-specific (*P* = 0.005) 5-year survival compared with segmentectomy in individuals with stage I adenocarcinoma (14). However, Dai et al. indicated that for T1aN0M0 NSCLC patients who were not suitable for lobectomy, segmentectomy should be recommended to those with tumors less than 2 cm in size (15). For patients who were not candidates for surgery, thermal ablation yielded better results for overall survival and acceptable local control. Mimae et al. reported that 3-year OS was slightly better after wedge resection than after segmentectomy plus lobectomy for patients over 80 years of age (89.4%, 95% CI, 73.8–95.9% vs. 75.8%, 95% CI, 62.0–85.2%; *P* = 0.14). Similarly, Willen et al. found that surgery was more common in the younger age group and that the use of stereotactic body radiotherapy (SBRT) increased with age (<69 years, 5.4%; >85 years, 35.8%) (16). Gender has also been shown to be an independent prognostic factor in surgically managed patients. Chansky et al. reported that female patients who underwent surgery alone had 5-year survival rates of 56.8%, higher than those of their male counterparts, whose 5-year survival rate was only 48.3% (*P* < 0.0001) (17). In conclusion, the selection of treatment should be made based on multiple factors after comprehensive evaluation.

When these factors were incorporated, our nomogram showed perfect discriminative ability. The C-indices were 0.704 (95% CI, 0.694–0.714) in the training cohort and 0.713 in the test cohort (95% CI, 0.697–0.728), respectively. These values are better than the C-index values obtained using TNM, which were 0.550 in the training set (95% CI, 0.408–0.683) and 0.548 in the test set (95% CI, 0.401–0.672, *P* < 0.001). The 3-year and 5-year validation curves also showed a high degree of agreement with the actual situation. In addition, the DCA curves revealed favorable potential clinical usefulness. Thus, all of the evaluations that were performed confirmed that our nomogram represents an excellent model that offers powerful prognostic performance in predicting OS and providing assistance with treatment decisions.

Several nomograms have been established for predicting the OS of NSCLC patients after surgery (18–20). However, no model that predicts the efficacy of specific treatments for previously untreated patients has been developed. Our model was designed to help doctors and patients choose the best treatment. Using our model, surgeons can calculate scores according to the patient’s physical condition and tumor characteristics and then provide treatment recommendations based on the predicted survival. Our model compares the influences of different treatment strategies on the prognosis of NSCLC patients, making it easily practicable and consistent with the actual situation that exists in lung cancer.

Although some previous studies demonstrated prognostic models for NSCLC, the results of those studies are slightly worse than ours. In 2019, Yuan et al. presented a nomogram for cancer-specific survival of patients with stage I NSCLC; that nomogram had a C-index of 0.64 (95% CI, 0.63–0.65) (21). The C-index reported in another study based on patients with stage I–III lung adenocarcinoma was only 0.69 (95% CI, 0.64–0.73) (22). The most important reason for the better result obtained in this study was the large sample size. The source of the population-based data used to establish and validate the model was the SEER program, which currently captures 40,000 cancer cases annually and stores cancer data for approximately 34.6% of the U.S. population in 18 SEER cancer registries. Our samples not only included different races and centers but could also be updated regularly, ensuring the timeliness of the model and minimizing selection bias.

Although many nomograms have been reported in the literature, few have been clinically applied. Possible reasons for this could be difficulty in obtaining specific data and high cost (21). David et al. (23) reported a quantitative-PCR-based assay for predicting survival in resectable lung cancer. Some models are based on radiomics (24) and artificial intelligence algorithms (25). These prediction models function well, but they are expensive to use and depend on variables that are not readily available in all clinical settings. The variables in our model are easily available, and this decreases the cost and increases the real-world practicality of its application.

This study has some limitations. First, because this was a retrospective research study, data selection bias was unavoidable. Second, cancer-specific survival (CSS) would be a more suitable metric than OS, but determination of the cause of death based on the information in the SEER database was not possible (26). Although we used multivariable analysis to reduce the impact of confounding, information on factors such as smoking, pulmonary function and gene mutation was not obtainable. Because the SEER database includes only patients in the United States, more than 85% of our cases involved white people. Therefore, the accuracy of predictions for the Asia–Pacific population has not been verified (27).

## Conclusion

We established a novel prognostic nomogram for predicting the OS of patients diagnosed with stage IA NSCLC using clinicopathological factors, tumor characteristics, and treatment modality. The nomogram has good discrimination and calibration ability. This model may be valuable in prognostic prediction and decision-making regarding treatment.

## Data availability statement

The original contributions presented in this study are included in the article/supplementary material, further inquiries can be directed to the corresponding authors.

## Ethics statement

Ethical review and approval was not required for the study on human participants in accordance with the local legislation and institutional requirements. Written informed consent from the patients or patients’ legal guardian/next of kin was not required to participate in this study in accordance with the national legislation and the institutional requirements.

## Author contributions

BX wrote the main manuscript text and validate the result. WY and ZZ wrote the review, edit, and supervised the manuscript text. ZY and ML managed data curation. CX and QW administrated the project, resources, and the investigation. LZ carried out the formal analysis and methodology. All authors contributed to the article and approved the submitted version.

## Footnotes

1
http://www.r-project.org
